# Biomarkers in Early Response to Brolucizumab on Pigment Epithelium Detachment Associated with Exudative Age-Related Macular Degeneration

**DOI:** 10.3390/biomedicines9060668

**Published:** 2021-06-10

**Authors:** Marco Rispoli, Chiara M. Eandi, Luca Di Antonio, Raphael Kilian, Andrea Montesel, Maria C. Savastano

**Affiliations:** 1 Chorioretinal Vasculopathies Unit, Surgery and Emergency Ophthalmology Department, Eye Hospital, 00136 Rome, Italy; rispolimarco@gmail.com; 2 Department of Ophthalmology, Jules Gonin Eye Hospital, Fondation Asile des Aveugles, University of Lausanne, 1002 Lausanne, Switzerland; andrea.montesel@gmail.com; 3 Department of Surgical Sciences, University of Torino, 10126 Torino, Italy; 4 UOC Ophthalmology and Surgery Department, ASL-1 Avezzano-Sulmona, 67051 L’Aquila, Italy; monsieurluca@yahoo.com; 5 Department of Neurosciences, Biomedicine and Movement Sciences, University of Verona, 37134 Verona, Italy; raphaelkilian8@yahoo.it; 6 Unit of Ophthalmology, Fondazione Policlinico A Gemelli, IRCCS, 00168 Rome, Italy; mariacristina.savastano@gmail.com; 7 Department of Ophthalmology, Università Cattolica Sacro Cuore, 00168 Rome, Italy

**Keywords:** age-related macular degeneration, innovative biotechnologies, Brolucizumab, exudative AMD, OCT angiography, personalized medicine

## Abstract

Background: The purpose of this study was to describe early changes in the morphology of pigment epithelium detachments (PED) after an intravitreal injection of Brolucizumab into eyes with macular neovascularization secondary to exudative age-related macular degeneration (e-AMD). Method: We included twelve eyes of 12 patients with PED secondary to e-AMD which were not responding to prior anti-VEGF treatments. An ophthalmic examination and an assessment of PED-horizontal maximal diameter (PED-HMD), PED-maximum high (PED-MH) and macular neovascularization (MNV) flow area (MNV-FA) by the means of structural optical coherence tomography (OCT) and OCT Angiography (OCT-A) were performed at baseline, as well as 1, 7, 14 and 30 days after the injection. Results: The mean age of the population of study was 78.4 (SD ± 4.8). The mean number of previous Ranibizumab or Aflibercept injections was 13 (SD ± 8). At the last follow-up visit, the PED-HMD did not significantly change (*p* = 0.16; F(DF:1.94, 20,85) = 1.9), the PED-MH showed a significant reduction [*p* = 0.01; F(DF:1.31, 14.13) = 6.84.] and the MNV-FA did not significantly differ (*p* = 0.1; F(1.97, 21.67) = 2.54) from baseline. No signs of ocular inflammation were observed during follow-up. Conclusions: A single Brolucizumab injection was able to determine the short-term effects on PEDs’ anatomical features of eyes with an unresponsive e-AMD.

## 1. Introduction

Worldwide, the incidence and prevalence of age-related macular degeneration (e-AMD) are relentlessly growing [1,2]. Over the age of 75, the risk of developing early and late AMD is 25% and 8%, respectively [3]. It was not a long time ago when the first anti-vascular endothelial growth factor (VEGF) drug received FDA approval for the treatment of e-AMD, and many steps forward have been made ever since [4]. As the median age of the general population keeps on growing, the burden associated with the treatment of AMD is expected to rise consistently. In fact, the overall number of patients with AMD is predicted to reach 288 million by 2040 [5]. The approval of Pegaptanib and previous positive experiences with Bevacizumab in colon cancer management fueled further research in the field of exudative AMD (e-AMD). In the following years, several randomized and non-randomized controlled trials have largely demonstrated the efficacy of different anti-VEGF molecules (i.e., Bevacizumab, Ranibizumab and Aflibercept) at contrasting macular neovascularization (MNV) [6,7,8]. Still, some of these trials and other real-world data made clear that many patients do not fully respond to current treatment regimens. A post hoc analysis of the VIEW studies showed that around 30% of patients receiving (q8w) Aflibercept-injections every 8 weeks still display some intra- or subretinal fluid 52 weeks after the beginning of the treatment [8]. Similarly, other studies showed that around 25% of the patients treated for e-AMD may be considered as both anatomical and functional non-responders [9,10,11]. The need for frequent injections and their high cost are responsible for a substantial social and economic burden on both patients and physicians [12].

Recently, a new humanized single-chain variable fragment was developed and named Brolucizumab (a.k.a. RTH258). By reducing its dimensions compared to the previous anti-VEGF drugs, this new agent promises to better penetrate the targeted retinal tissues, to reduce immunogenicity and to provide a longer durability [4]. Particularly, the latter has been confirmed by two recent randomized clinical trials, where more than half of the enrolled patients were able to have their neovascularization controlled by a three-monthly (q12w) treatment regimen [13]. This allows one to decrease the treatment burden and to improve patients’ compliance [14]. Another important advantage this drug displays is its high drying effect. Despite the final best corrected visual acuity (BCVA) being similar to that obtained with q8w. Aflibercept, both after 16 and 48 weeks, Brolucizumab obtained a significantly lower rate of disease activity (measured by central subfield thickness and by the presence of intra or subretinal fluid) [12]. Although some severe side-effects have been reported [15], these seem to be rare and there is evidence supporting the safety of Brolucizumab [13,16]. Further, the short-term real-world outcomes reported in the SHIFT study demonstrated that the switch to Brolucizumab may represent a good option in patients with poorly responsive AMDs [17].

Parallel to the surge of anti-VEGFs and the constant research for other new therapeutic approaches [18] in the last decade, optical coherence tomography (OCT) has gained a crucial role in the management of retinal diseases. Indeed, structural OCT allows for an in vivo analysis of the finest morphological retinal details [19]. Identifying each patients’ disease characteristics is essential in order to define the best patient-tailored treatment possible. As reported in the pioneering work of Schmidt-Erfurth et al., inter-individual treatment requirements are vastly heterogeneous, and there is a need to build predictive models based on automated image analysis of OCT scans in order to address these [19]. Using these models, artificial intelligence (AI) will probably play a major role in future ophthalmological clinical practice.

The introduction of optical coherence tomography angiography (OCT-A) has recently fueled the interest in retinal vascular diseases. OCT-A allows for direct visualization of the retinal and choroidal vascularization without the need for an intravenous dye injection. Despite having some limitations, this technique grants a fast and sensible detection of any changes in vessel morphology and overall neovascular-disease activity [20,21]. With the introduction of Brolucizumab being fairly recent, and the diffusion of OCT-A still not at its peak, there is a lack of studies analyzing the effects this drug determines on MNV using the latter technique.

Retinal pigment epithelium detachments (PED), which represent the separation between the basal lamina of the retinal pigment epithelium (RPE) and the inner collagenous layers of Bruch’s membrane, are considered to be prominent features of AMD. Three main types of PED have been described: serous, drusenoid and fibrovascular detachments. The latter has been classically defined by its angiographic and tomographic appearance, as characterized by a stippled granular hyperfluorescence and by the presence of a deep area of backscattering within the PED itself. Additionally, fibrovascular PEDs have always been considered to represent a subset of occult choroidal neovascular membranes (CNV) [21]. In recent years, huge steps forward have been made in our understanding of retinal dystrophies and of RPE’s role in the development of CNVs [22,23,24,25]. However, our knowledge about the exact pathophysiology of PEDs is still incomplete. This seems to be related to the accumulation of fluid and debris due to the malfunction of a degenerated Bruch’s membrane [26].

Since there seems to be a correlation between their morphological structure and their evolution to exudative AMD, the interest in PEDs’ pathogenesis and their prognostic value has risen as of late.

To our knowledge, there is currently no study reporting the effects of Brolucizumab on PEDs associated with a non-responding e-AMD.

By using structural OCT and OCT-A imaging in this study, we aimed to assess the early changes in pigment epithelium detachment morphology after intravitreal injection of Brolucizumab into eyes with MNVs.

## 2. Methods

This study was conducted in agreement with the tenets of the Declaration of Helsinki for research involving human subjects, and was approved by Institutional Review Committees (ID number: 3680 approved on 19 January 2021). Written informed consent to participate in this observational study was obtained from all patients. Inclusion criteria were (1) a diagnosis of PED secondary to e-AMD, (2) having undergone a Brolucizumab (Beovu^®^, Novertis, Basel, Switzerland) injection, (3) the presence of high-quality structural spectral domain OCT (SD-OCT) and OCT-A images (i.e., signal strength index higher than 7/10) not affected by artifacts and (4) not being responsive (i.e., absence of any sign of exudation regression) to other intravitreal anti-VEGFs. Exclusion criteria were the concomitant presence of ophthalmological diseases potentially able to confound the interpretation of images (e.g., diabetic retinopathy, glaucoma, chorioretinal diseases), the lack of high-quality images and exudation regression following prior anti-VEGF treatments. All enrolled patients underwent a comprehensive ophthalmological evaluation. This included measurement of the BCVA using the early treatment diabetic retinopathy study (ETDRS) charts and the logarithm of the minimum angle of resolution (LogMAR) system, a dilated fundus examination and the performance of OCT and OCT-A scans. The latter was performed using the RTVue XR Avanti OCT-A system (AngioVue system, Optovue^®^ Inc., Fremont, CA, USA). Imaging of the retinal lesions and analysis of the lesions’ development were repeated the day after the injection, and then 7, 14 and 30 days thereafter.

### 2.1. PED Quantitative Assessment

Both the PED-horizontal maximal diameter (PED-HMD) and the PED-maximum high (PED-MH) were manually measured using the caliper tool available on all SD-OCT devices.

**Figure 1:**
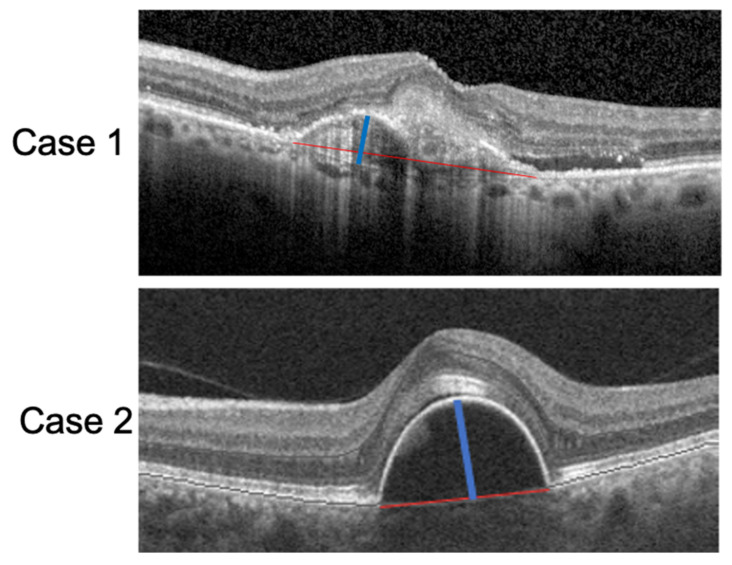
SD-OCT scan of two patients with a PED. The red lines correspond to the PED-HMDs and the blue lines to the PED-MHs.

All measurements were assessed by two independent retinal specialists (M.C.S and R.K) and compared using the Cohen’s kappa coefficient. The interobserver agreement on image analysis was 0.92 (k = 0.225, *p* < 0.01). Examples of PED-HMD and PED-MH are reported in Figure 1.

### 2.2. OCT-A and Neovascularization Assessment

**Figure 2:**
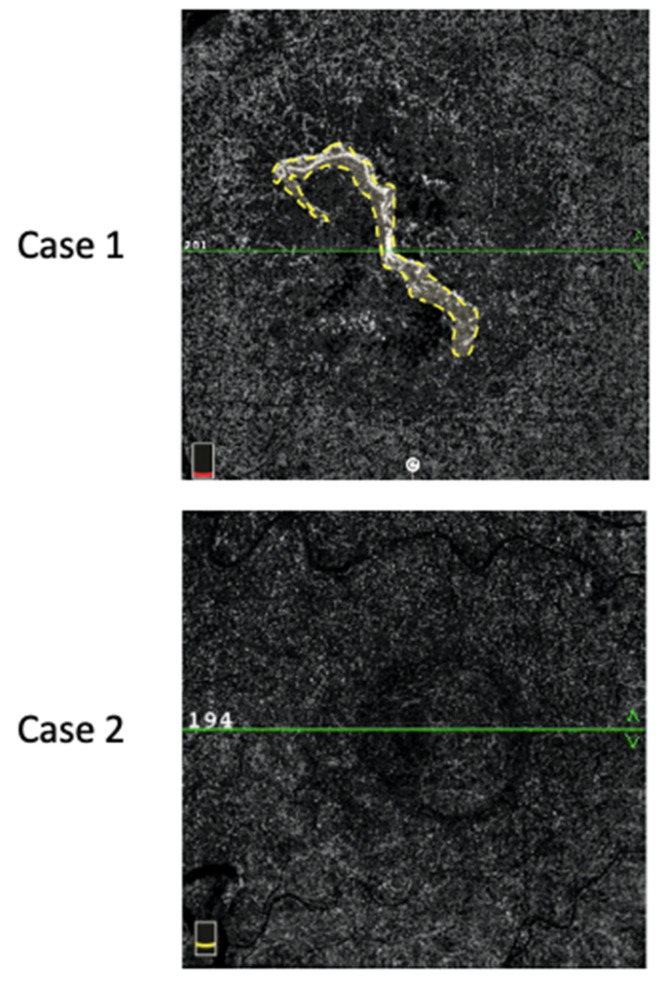
MNV-flow area (mm^2^) defined by the yellow dotted line (Case 1). In Case 2, no flow could be detected inside the PED; thus, the flow area was considered null.

MNV flow areas were assessed using a semiautomatic algorithm commercially available on the OCT-A device. In cases of PEDs with hyporeflective contents not allowing for a clear visualization of the MNV, we considered the value of the MNV-flow area to be “0”. An example of MNV-flow area measurement is shown in Figure 2.

### 2.3. Statistical Analysis

The sample size was calculated via an a priori sample size estimation method using the G-Power software package (version 3.1.9.6, Universität Kiel, Germany). Assuming a minimum difference of 15%, a residual standard deviation of 10%, a power of 0.08 and an alpha of 0.05 to highlight the effect, the required sample size was 12 patients. Data were analyzed with the GraphPad PRISM Software (v.8.0; GraphPad, La Jolla, CA, USA). Quantitative variables were presented as mean ± SD and were evaluated using the analysis of variance (ANOVA) for repeated measures. A pairwise post hoc analysis using Holm–Sidak’s correction for multiple comparisons test was also performed. A *p* value less than 0.05 was considered statistically significant.

## 3. Results

Twelve eyes of 12 patients that did not respond to previous anti-VEGF treatments were enrolled in the study. Five eyes had previously undergone Ranibizumab or Aflibercept injections. Seven eyes previously received both Aflibercept and Ranibizumab injections. Table 1 reports baseline population characteristics.

At baseline, the mean PED-HMD and PED-MH were 1548.36 µm (±1041.96) and 207.36 µm (±82.89), respectively. The mean MNV-flow area was 0.25 mm^2^ (±0.45) and the mean BCVA was 0.48 LogMAR (±0.42). MNV-flow area was found to be undetectable on OCT-A scans of 4 out of 12 eyes (33%). 

Details of considered measurements across the different follow-up time points are reported in Table 2. All included eyes (100%) displayed some mixture of intraretinal fluid (IRF) and subretinal fluid (SRF) concomitant with the PED. None displayed macular hemorrhages.

**Figure 3:**
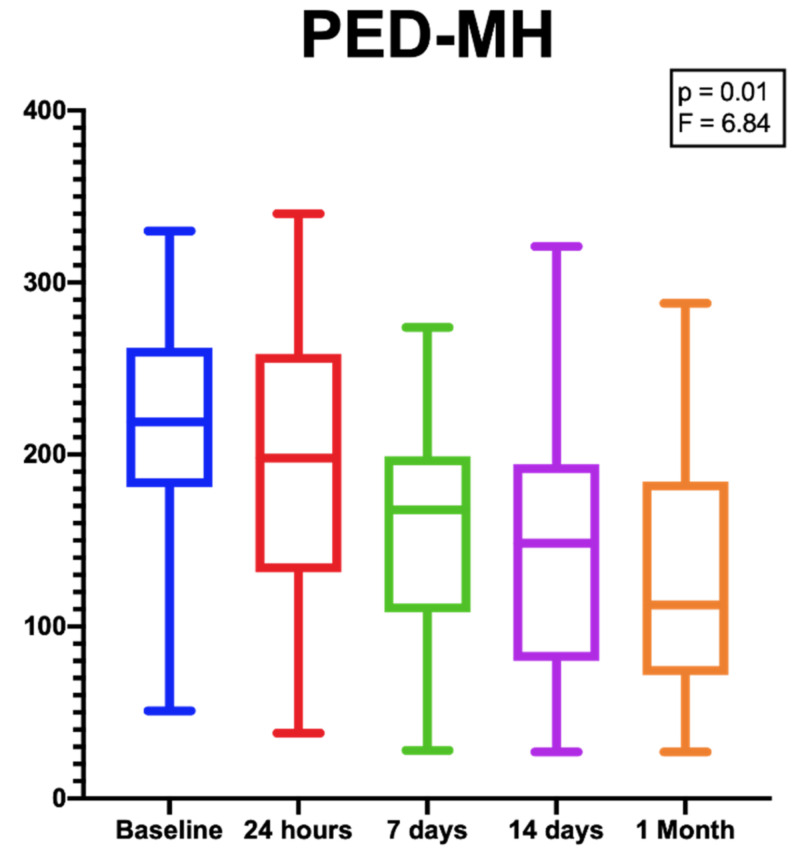
Box plots showing the significant reduction in PED-MHs from baseline to the end of the follow-up period. [*p* = 0.01; F (DF:1.31, 14.13) = 6.84.].

Despite the difference in PED-HMDs between baseline and 1 month after the injection not being statistically significant [*p* = 0.16; F (DF: 1.94, 20,85) = 1.9], the PED-MHs showed a significant reduction during the same follow-up period [*p* = 0.01; F (DF:1.31, 14.13) = 6.84.] (Figure 3). 

**Figure 4:**
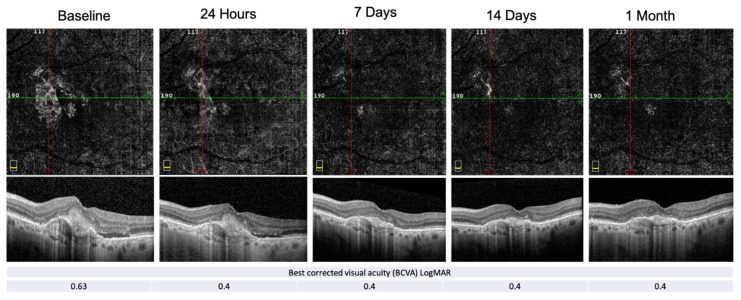
E-AMD morpho-functional assessment by structural OCT, OCT-A and BCVA. Structural OCT shows the reabsorption of the subretinal hyper-reflective material (SHRM) and the resolution of the subretinal fluid at the end of the follow-up period. PED-HMDs did not significantly change during the visits, whereas the PED-MH did. The red and green line show respectively the vertical and horizontal scan position.

An example of PEDs’ anatomical and functional fluctuation during the follow-up period is shown in Figure 4.

The MNV flow-area failed to show a significant reduction over the observation period [*p* = 0.1; F (DF:1.97, 21.67) = 2.54]; although, as previously specified, at baseline, we were unable to identify any flow area in 4 of the 12 examined eyes.

None of the included eyes displayed a complete disappearance of the MNV after the injection.

Finally, the BCVA values at the end of the follow-up period were not significantly different from baseline [*p* = 0.58; F (2.23, 24.53) = 0.58].

While the PED-MH showed a continuous reduction during the whole period of observation, the PED-HMDs displayed an increase at the 7-day timepoint. This, however, was not statistically significant.

No signs of ocular inflammation (neither in the anterior nor in the posterior segment) and no other complications were ever observed after the injection of Brolucizumab.

At the end of the follow-up period, all the treated eyes (100%) had their IRF/SRF completely reabsorbed. 

## 4. Discussion

One of the main goals of medical research is to find diseases’ biomarkers and prognostic factors. This consideration also suits the field of ophthalmology, especially since multimodal imaging methods have become essential diagnostic tools in everyday clinical practice [27,28,29]. Nowadays, OCT and OCT-A play a crucial role in both the diagnosis and the follow-up of patients with retinal affections. This is particularly true for patients with both dry and wet AMD [30].

In the former, multimodal imaging is essential for a correct diagnosis and for the assessment of possible complications (e.g., the simultaneous onset of neovascularization). The performance of fundus autofluorescence (AF) and enface scans are of particular importance in order to follow patients with atrophic AMDs. In fact, these techniques can easily identify and help predict any increase in the area of retinal atrophy [31,32]. The role of an abnormal choriocapillaris perfusion of the macular area also has been recently reported to be a contributing factor to the progression of non-exudative AMD [33]. The investigation of retinal pigment epithelial and outer retinal atrophy (RORA) is continuously developing and can be considered a prognostic factor for atrophy progression or change to an exudative form of AMD [29,34,35].

Despite the recent technological progress and the improvements in our knowledge of the disease, interpreting the clinical course of exudative AMD remains challenging. The search for new diagnostic biomarkers and for signs able to predict the reactivation of the pathology remains the main goal of retinal clinicians interested in wet AMD. As time goes on, finding these diseases’ biomarkers through multimodal imaging is crucial in order to introduce artificial intelligence in the clinical practice [36]. The latter would represent a huge advantage for both patients and physicians.

The detection of new possible prognostic factors is also important for understanding the response to our therapies. In vivo histological analysis using OCT scans is probably the most attractive method for studying the disease through artificial intelligence. In fact, these scans are rich in details and can be easily accessed by radiomics [37,38]. Moreover, structural OCT and OCT-A are amongst the most effective non-invasive imaging tools for several retinal diseases. As a matter of fact, in some cases, these techniques are even considered to be the first-line diagnostic approaches over the older dye imaging methods [39,40,41]. 

PEDs’ morphological features have been previously described to be useful for differentiating and examining some frequently diagnosed clinical entities, such as AMD [42]. In a recent study by Lupidi et al. [43], the authors reported that PEDs’ SD-OCT-appearance might be considered as a phenotypic prognostic factor for quiescent MNV. Particularly, they found the growth in PEDs’ greatest linear diameter to be associated with the maintenance of a quiescent phenotype, whereas the growth in PEDs’ maximal height was associated more frequently with the development of an exudative phenotype. The latter was confirmed by studies by Lam et al. [44] and Serra et al. [43]. These suggest that PEDs’ “contour” morphology might be indicative of a possible future exudative development. Flat, “wrinkled” PEDs are more prone to quiescence than smooth, peaked ones.

Although previous studies suggest the reduction in the flow area might be considered a potential biomarker to assess MNV responsiveness to anti-VEGFs, there are some controversies, since OCT-A has a variable sensitivity [20,45,46]. 

As an early response to a single intravitreal Brolucizumab injection in our study, PEDs showed a significant reduction in their maximal heights [*p* = 0.01; F = (DF:1.31, 14.13) 6.84.], whereas no significant difference was found in terms of horizontal maximal diameter, flow area and BCVA. In the phase 3 Hawk and Harrier trials, 4 weeks after the injection, the authors showed an increase in BCVA of about four letters [12]. However, these studies included only naïve patients with a BCVA between 20/32 and 20/400, also excluding those with macular fibrosis or geographic atrophy. The poor visual acuity results obtained in our study may be related to both the short-term follow-up and the chronic nature of the included patients’ disease. The latter may have induced an irreversible degeneration of the photoreceptors. With regard to the lack of improvement in the MNV-flow area, it has to be specified that, at baseline, we were able to detect a flow area in only 77% (8/12) of the included eyes. Despite not being statistically significant, we actually did show a decrease in the mean flow area (i.e., from 0.25 mm^2^ (±0.45) at baseline, to 0.12 mm^2^ (±0.33) 1 month after the injection). The lack of significance might be related to the small number of study participants. Overall, the literature seems to agree that the injection of anti-VEGFs usually leads to a reduction in the mean MNV-associated flow area [45,47]. A complete regression of the MNV after the injection never occurred in our series. In fact, during follow-up, we were always able to detect a minimum flow on OCT-A scans. This finding is in agreement with the arterialization sign described by Spaide et al. Despite previous anti-VEGF treatments, the main trunk of the new vessel is always left behind [48]. 

One month after the injection, all of the included eyes showed a complete reabsorption of both the SRF and the IRF they displayed at baseline. This further proves the high drying effect that Brolucizumab has compared to other anti VEGF agents. 

Finally, none of the included patients developed any kind of adverse events. This was a significant finding, even though we observed our patients for only a limited period of time. Previously reported incidences of ocular inflammation and retinal artery occlusion have been as high as 2% and 0.6%, respectively. Such complications might have required a larger cohort of patients in order to be detected [13].

The lack of detection of low flow rates still represents a major limitation in OCT-A technology [49]; however, the continuous evolution of new algorithms will soon allow us to solve this problem. The wide variability in flow-sensibility among different types of OCT-A devices should be kept in mind when evaluating patients in everyday clinical practice [50]. 

Major limitations of our investigation were its limited sample size and its short period of observation. In the future, we commit to reporting the long-term follow-up outcomes of this study. Additionally, we did not include any evaluation of the influence Brolucizumab might have on vitreal proteomic and RPE’s genetic expression. The latter might be an interesting point to investigate in the future. 

Moreover, our results referred to a cohort of patients that had been previously treated with numerous anti-VEGF injections, and therefore cannot be applied to patients with naïve e-AMDs.

## 5. Conclusions

In this study, we demonstrated the significant short-term effects of a single Brolucizumab injection at reducing PEDs’ vertical dimensions in patients with an unresponsive e-AMD. Further research should be carried out aiming to identify the advantages of the newest anti-VEGF treatments and the possible biomarkers of e-AMD-responsiveness to the latter.

## Figures and Tables

**Table 1:** Characteristics of the population of study and details before the Brolucizumab injection.

Mean age, years (SD)	78.4 (±4.8)
gender, n (%)	8/4 (F/M)
Number of previous anti-VEGF injections	13 (±8) (Ranibizumab or Aflibercept)

**Table 2:** Characteristics of structural OCT and OCT-A at baseline, after 24 h, at 7 days, at 14 days and 1 month after the Brolucizumab injection.

	PED-HMD (±SD) µm	PED-MH (±SD) µm	OCTA MNV-FA (mm^2)^	BCVA (LogMAR)
*Baseline*	1548.36 (±1041.96)	207.36 (±82.89)	0.25 (±0.45)	0.48 (±0.42)
*24 h*	1588.41 (±897.41)	195.75 (±85.65)	0.22 (±0.41)	0.44 (±0.33)
*7 days*	1638.72 (±1022.77)	163.25 (±69.53)	0.17 (±0.37)	0.49 (±0.40)
*14 days*	1398.66 (±959.67)	145.66 (±80.34)	0.12 (±0.34)	0.48 (±0.41)
*1 Month*	1322.63 (±905.88)	127.5 (±77.79)	0.12 (±0.33)	0.46 (±0.41)

PED: pigment epithelium detachment; PED-HMD: pigment epithelium detachment-horizontal maximal diameter; PED-MH: pigment epithelium detachment-maximum high; OCTA: optical tomography angiography; MNV-FA: macular neovascularization-flow area; LogMAR: logarithm of the minimum angle of resolution.

## Data Availability

The data presented in this study are available on request from the corresponding author. The data are not publicly available due to specific regulations of the centers.

## References

[1] Owen C.G., Jarrar Z., Wormald R., Cook D.G., Fletcher A.E., Rudnicka A.R. (2012). The Estimated Prevalence and Incidence of Late Stage Age Related Macular Degeneration in the UK. Br. J. Ophthalmol..

[2] Sedeh F.B., Scott D.A.R., Subhi Y., Sørensen T.L. (2017). Prevalence of Neovascular Age-Related Macular Degeneration and Geographic Atrophy in Denmark. Dan. Med. J..

[3] Thomas C.J., Mirza R.G., Gill M.K. (2021). Age-Related Macular Degeneration. Med. Clin. N. Am..

[4] Gragoudas E.S., Adamis A.P., Cunningham E.T., Feinsod M., Guyer D.R. (2004). VEGF Inhibition Study in Ocular Neovascularization Clinical Trial Group Pegaptanib for Neovascular Age-Related Macular Degeneration. N. Engl. J. Med..

[5] Wong W.L., Su X., Li X., Cheung C.M.G., Klein R., Cheng C.-Y., Wong T.Y. (2014). Global Prevalence of Age-Related Macular Degeneration and Disease Burden Projection for 2020 and 2040: A Systematic Review and Meta-Analysis. Lancet Glob. Health.

[6] Rosenfeld P.J., Brown D.M., Heier J.S., Boyer D.S., Kaiser P.K., Chung C.Y., Kim R.Y., MARINA Study Group (2006). Ranibizumab for Neovascular Age-Related Macular Degeneration. N. Engl. J. Med..

[7] Martin D.F., Maguire M.G., Ying G., Grunwald J.E., Fine S.L., Jaffe G.J., CATT Research Group (2011). Ranibizumab and Bevacizumab for Neovascular Age-Related Macular Degeneration. N. Engl. J. Med..

[8] Heier J.S., Brown D.M., Chong V., Korobelnik J.-F., Kaiser P.K., Nguyen Q.D., Kirchhof B., Ho A., Ogura Y., Yancopoulos G.D. (2012). Intravitreal Aflibercept (VEGF Trap-Eye) in Wet Age-Related Macular Degeneration. Ophthalmology.

[9] Holz F.G., Tadayoni R., Beatty S., Berger A., Cereda M.G., Cortez R., Hoyng C.B., Hykin P., Staurenghi G., Heldner S. (2015). Multi-Country Real-Life Experience of Anti-Vascular Endothelial Growth Factor Therapy for Wet Age-Related Macular Degeneration. Br. J. Ophthalmol..

[10] Amoaku W.M., Chakravarthy U., Gale R., Gavin M., Ghanchi F., Gibson J., Harding S., Johnston R.L., Kelly S.P., Kelly S. (2015). Defining Response to Anti-VEGF Therapies in Neovascular AMD. Eye.

[11] Krebs I., Glittenberg C., Ansari-Shahrezaei S., Hagen S., Steiner I., Binder S. (2013). Non-Responders to Treatment with Antagonists of Vascular Endothelial Growth Factor in Age-Related Macular Degeneration. Br. J. Ophthalmol..

[12] Dugel P.U., Jaffe G.J., Sallstig P., Warburton J., Weichselberger A., Wieland M., Singerman L. (2017). Brolucizumab Versus Aflibercept in Participants with Neovascular Age-Related Macular Degeneration: A Randomized Trial. Ophthalmology.

[13] Dugel P.U., Koh A., Ogura Y., Jaffe G.J., Schmidt-Erfurth U., Brown D.M., Gomes A.V., Warburton J., Weichselberger A., Holz F.G. (2020). HAWK and HARRIER: Phase 3, Multicenter, Randomized, Double-Masked Trials of Brolucizumab for Neovascular Age-Related Macular Degeneration. Ophthalmology.

[14] Ferro Desideri L., Traverso C.E., Nicolò M. (2021). Brolucizumab: A Novel Anti-VEGF Humanized Single-Chain Antibody Fragment for Treating w-AMD. Expert Opin. Biol. Ther..

[15] Jain A., Chea S., Matsumiya W., Halim M.S., Yaşar Ç., Kuang G., Sepah Y.J., Khanani A.M., Do D.V., Nguyen Q.D. (2020). Severe Vision Loss Secondary to Retinal Arteriolar Occlusions after Multiple Intravitreal Brolucizumab Administrations. Am. J. Ophthalmol. Case Rep..

[16] Avaylon J., Lee S., Gallemore R.P. (2020). Case Series on Initial Responses to Intravitreal Brolucizumab in Patients with Recalcitrant Chronic Wet Age-Related Macular Degeneration. Int. Med. Case Rep. J..

[17] Bulirsch L.M., Saßmannshausen M., Nadal J., Liegl R., Thiele S., Holz F.G. (2021). Short-Term Real-World Outcomes Following Intravitreal Brolucizumab for Neovascular AMD: SHIFT Study. Br. J. Ophthalmol..

[18] Solanki A., Smalling R., Parola A.H., Nathan I., Kasher R., Pathak Y., Sutariya V. (2019). Humanin Nanoparticles for Reducing Pathological Factors Characteristic of Age-Related Macular Degeneration. Curr. Drug Deliv..

[19] Romo-Bucheli D., Erfurth U.S., Bogunovic H. (2020). End-to-End Deep Learning Model for Predicting Treatment Requirements in Neovascular AMD From Longitudinal Retinal OCT Imaging. IEEE J. Biomed. Health Inform..

[20] Faatz H., Farecki M.-L., Rothaus K., Gunnemann F., Gutfleisch M., Lommatzsch A., Pauleikhoff D. (2019). Optical Coherence Tomography Angiography of Types 1 and 2 Choroidal Neovascularization in Age-Related Macular Degeneration during Anti-VEGF Therapy: Evaluation of a New Quantitative Method. Eye.

[21] Sulzbacher F., Pollreisz A., Kaider A., Kickinger S., Sacu S., Schmidt-Erfurth U., Vienna Eye Study Center (2017). Identification and Clinical Role of Choroidal Neovascularization Characteristics Based on Optical Coherence Tomography Angiography. Acta Ophthalmol..

[22] Donato L., Scimone C., Alibrandi S., Pitruzzella A., Scalia F., D’Angelo R., Sidoti A. (2020). Possible A2E Mutagenic Effects on RPE Mitochondrial DNA from Innovative RNA-Seq Bioinformatics Pipeline. Antioxidants.

[23] Scimone C., Alibrandi S., Scalinci S.Z., Trovato Battagliola E., D’Angelo R., Sidoti A., Donato L. (2020). Expression of Pro-Angiogenic Markers Is Enhanced by Blue Light in Human RPE Cells. Antioxidants.

[24] Donato L., Scimone C., Alibrandi S., Abdalla E.M., Nabil K.M., D’Angelo R., Sidoti A. (2020). New Omics-Derived Perspectives on Retinal Dystrophies: Could Ion Channels-Encoding or Related Genes Act as Modifier of Pathological Phenotype?. Int. J. Mol. Sci..

[25] Donato L., Abdalla E.M., Scimone C., Alibrandi S., Rinaldi C., Nabil K.M., D’Angelo R., Sidoti A. (2021). Impairments of Photoreceptor Outer Segments Renewal and Phototransduction Due to a Peripherin Rare Haplotype Variant: Insights from Molecular Modeling. Int. J. Mol. Sci..

[26] Zayit-Soudry S., Moroz I., Loewenstein A. (2007). Retinal Pigment Epithelial Detachment. Surv. Ophthalmol..

[27] Sato T., Suzuki M., Ooto S., Spaide R.F. (2015). Multimodal Imaging Findings and Multimodal Vision Testing in Neovascular Age-Related Macular Degeneration. Retina.

[28] Sacconi R., Baldin G., Carnevali A., Querques L., Rabiolo A., Marchini G., Bandello F., Querques G. (2018). Response of Central Serous Chorioretinopathy Evaluated by Multimodal Retinal Imaging. Eye.

[29] Guymer R.H., Rosenfeld P.J., Curcio C.A., Holz F.G., Staurenghi G., Freund K.B., Schmitz-Valckenberg S., Sparrow J., Spaide R.F., Tufail A. (2020). Incomplete Retinal Pigment Epithelial and Outer Retinal Atrophy in Age-Related Macular Degeneration: Classification of Atrophy Meeting Report 4. Ophthalmology.

[30] Nassisi M., Sadda S.R. (2021). Ocular Imaging for Enhancing the Understanding, Assessment, and Management of Age-Related Macular Degeneration. Adv. Exp. Med. Biol..

[31] Bandello F., Sacconi R., Querques L., Corbelli E., Cicinelli M.V., Querques G. (2017). Recent Advances in the Management of Dry Age-Related Macular Degeneration: A Review. F1000Research.

[32] Vogl W.-D., Bogunović H., Waldstein S.M., Riedl S., Schmidt-Erfurth U. (2021). Spatio-Temporal Alterations in Retinal and Choroidal Layers in the Progression of Age-Related Macular Degeneration (AMD) in Optical Coherence Tomography. Sci. Rep..

[33] Shi Y., Zhang Q., Zhou H., Wang L., Chu Z., Jiang X., Shen M., Thulliez M., Lyu C., Feuer W. (2021). Correlations Between Choriocapillaris and Choroidal Measurements and the Growth of Geographic Atrophy Using Swept Source OCT Imaging. Am. J. Ophthalmol..

[34] Savastano M.C., Falsini B., Cozzupoli G.M., Savastano A., Gambini G., De Vico U., Minnella A.M., Placidi G., Piccardi M., Rizzo S. (2020). Retinal Pigment Epithelial and Outer Retinal Atrophy in Age-Related Macular Degeneration: Correlation with Macular Function. J. Clin. Med..

[35] Corvi F., Corradetti G., Tiosano L., McLaughlin J.A., Lee T.K., Sadda S.R. (2021). Topography of Choriocapillaris Flow Deficit Predicts Development of Neovascularization or Atrophy in Age-Related Macular Degeneration. Graefe’s Arch. Clin. Exp. Ophthalmol..

[36] Grechenig C., Reiter G.S., Riedl S., Arnold J., Guymer R., Gerendas B.S., Bogunović H., Schmidt-Erfurth U. (2021). Impact of Residual Subretinal Fluid Volumes on Treatment Outcomes in a SRF-Tolerant Treat & Extend Regimen. Retina.

[37] Banerjee I., de Sisternes L., Hallak J.A., Leng T., Osborne A., Rosenfeld P.J., Gregori G., Durbin M., Rubin D. (2020). Prediction of Age-Related Macular Degeneration Disease Using a Sequential Deep Learning Approach on Longitudinal SD-OCT Imaging Biomarkers. Sci. Rep..

[38] Wang J., Hormel T.T., Gao L., Zang P., Guo Y., Wang X., Bailey S.T., Jia Y. (2020). Automated Diagnosis and Segmentation of Choroidal Neovascularization in OCT Angiography Using Deep Learning. Biomed. Opt. Express.

[39] Savastano M.C., Rispoli M., Lumbroso B., Di Antonio L., Mastropasqua L., Virgili G., Savastano A., Bacherini D., Rizzo S. (2020). Fluorescein Angiography versus Optical Coherence Tomography Angiography: FA vs OCTA Italian Study. Eur. J. Ophthalmol..

[40] Falsini B., Placidi G., De Siena E., Savastano M.C., Minnella A.M., Maceroni M., Midena G., Ziccardi L., Parisi V., Bertelli M. (2021). USH2A-Related Retinitis Pigmentosa: Staging of Disease Severity and Morpho-Functional Studies. Diagnostics.

[41] Barca F., Bacherini D., Dragotto F., Tartaro R., Lenzetti C., Finocchio L., Virgili G., Caporossi T., Giansanti F., Savastano A. (2020). OCT Angiography Findings in Macula-ON and Macula-OFF Rhegmatogenous Retinal Detachment: A Prospective Study. J. Clin. Med..

[42] Lumbroso B., Savastano M.C., Rispoli M., Balestrazzi A., Savastano A., Balestrazzi E. (2011). Morphologic Differences, According to Etiology, in Pigment Epithelial Detachments by Means of En Face Optical Coherence Tomography. Retina.

[43] Serra R., Coscas F., Boulet J.F., Cabral D., Lupidi M., Coscas G.J., Souied E.H. (2020). Predictive Activation Biomarkers of Treatment-Naive Asymptomatic Choroidal Neovascularization in Age-Related Macular Degeneration. Retina.

[44] Lam D., Semoun O., Blanco-Garavito R., Jung C., Nguyen D.T., Souied E.H., Mimoun G. (2018). Wrinkled Vascularized Retinal Pigment Epithelium Detachment Prognosis after Intravitreal Anti-Vascular Endothelial Growth Factor Therapy. Retina.

[45] Di Antonio L., Toto L., Mastropasqua A., Brescia L., Erroi E., Lamolinara A., Di Nicola M., Mastropasqua L. (2018). Retinal Vascular Changes and Aqueous Humor Cytokines Changes after Aflibercept Intravitreal Injection in Treatment-Naïve Myopic Choroidal Neovascularization. Sci. Rep..

[46] Cabral D., Coscas F., Pereira T., Français C., Geraldes C., Laiginhas R., Rodrigues C., Kashi A.K., Nogueira V., Falcão M. (2020). Quantitative Optical Coherence Tomography Angiography Biomarkers in a Treat-and-Extend Dosing Regimen in Neovascular Age-Related Macular Degeneration. Transl. Vis. Sci. Technol..

[47] Cennamo G., Amoroso F., Schiemer S., Velotti N., Alfieri M., de Crecchio G. (2019). Optical Coherence Tomography Angiography in Myopic Choroidal Neovascularization after Intravitreal Ranibizumab. Eur. J. Ophthalmol..

[48] Spaide R.F. (2015). Optical Coherence Tomography Angiography Signs of Vascular Abnormalization With Antiangiogenic Therapy for Choroidal Neovascularization. Am. J. Ophthalmol..

[49] Tan A.C.S., Freund K.B., Balaratnasingam C., Simhaee D., Yannuzzi L.A. (2018). Imaging of Pigment Epithelial Detachments with Optical Coherence Tomography Angiography. Retina.

[50] Siggel R., Spital C., Lentzsch A., Liakopoulos S. (2020). Comparison of Automated versus Manually Modified OCT Angiography En Face Slabs for Detection of Choroidal Neovascularization. Ophthalmol. Retina.

